# Initial Results From SOMMA: Contribution of Mitochondrial Function to Walking and Fitness

**DOI:** 10.1093/geroni/igab046.481

**Published:** 2021-12-17

**Authors:** Steve Cummings, Peggy Cawthon, Bret Goodpaster, Russell Hepple, Nancy W Glynn, Stephen Kritchevsky, Anne Newman, Paul Coen

**Affiliations:** 1 California Pacific Medical Center, San Francisco, California, United States; 2 AdventHealth, Orlando, Florida, United States; 3 University of Florida, Gainsville, Florida, United States; 4 University of Pittsburgh Graduate School of Public Health, Pittsburgh, Pennsylvania, United States; 5 Wake Forest School of Medicine, Winston Salem, North Carolina, United States; 6 University of Pittsburgh, Pittsburgh, Pennsylvania, United States

## Abstract

We hypothesize that the capacity of mitochondria in quadriceps skeletal muscle to generate ATP energy by respirometry (OXPHOS) in biopsies from the vastus lateralis, and in whole quadriceps muscle by 31PMRS (ATPmax) would contribute to 4 and 400m gait speed and to peak oxygen consumption on treadmill testing (VO2peak). In analyses from the first SOMMA participants recruited (N=122), OXPHOS was similarly associated with 4m (r=0.21) and 400 m (r=0.21) walking speed (P<0.01). However, ATPmax was not associated with either 4m or 400m walking speed (r=-0.02 and -0.07 respectively). In contrast both OXPHOS (r=0.43) and ATP max (r=0.35) were more strongly correlated with fitness (VO2 peak). These findings suggest that in older people, the mitochondrial capacity to generate ATP plays an important role walking speed and may be even more important to fitness.

